# A 13-plex of tetra- and penta-STRs to identify zebrafish

**DOI:** 10.1038/s41598-020-60842-5

**Published:** 2020-03-02

**Authors:** Patrick J. Venta, Anthony K. Nguyen, Marie-Claude Senut, William G. Poulos, Sukumal Prukudom, Jose B. Cibelli

**Affiliations:** 1 Microbiology & Molecular Genetics, Michigan State University, East Lansing, MI 48823 USA; 2 Small Animal Clinical Sciences, College of Veterinary Medicine, Michigan State University, East Lansing, MI 48823 USA; 3 Biomilab LLC, Lansing, MI 48910 USA; 4 Animal Science, College of Agriculture and Natural Resources, Michigan State University, East Lansing, MI 48823 USA; 5 Center for Advanced Studies for Agriculture and Food, Kasetsart University Institute for Advanced Studies, Kasetsart University (CASAF, NRU-KU), Bangkok, 10900 Thailand; 6 Large Animal Clinical Sciences, College of Veterinary Medicine, Michigan State University, East Lansing, MI 48823 USA

**Keywords:** Biological techniques, Sequencing, DNA sequencing, Genetics, Genetic markers

## Abstract

The zebrafish species *Danio rerio* has become one of the major vertebrate model organisms used in biomedical research. However, there are aspects of the model that need to be improved. One of these is the ability to identify individual fish and fish lines by DNA profiling. Although many dinucleotide short tandem repeat (diSTR) markers are available for this and similar purposes, they have certain disadvantages such as an excessive polymerase slippage (“stutter”) that causes difficulties in automated genotyping and cross-laboratory comparisons. Here we report on the development of a 13-plex of tetranucleotide and pentanucleotide STRs (tetraSTRs and pentaSTRs, respectively) that have low stutter. The system uses an inexpensive universal primer labelling system, which can easily be converted to a direct labeling system if desired. This 13-plex was examined in three zebrafish lines (NHGRI-1, kca33Tg, and kca66Tg, originally obtained from ZIRC). The average observed heterozygosity (Ho) and expected heterozygosity (He) in these highly inbred lines were 0.291 and 0.359, respectively, which is very similar to what has been found with diSTRs. The probability of identity (PI) for all fish tested was 2.1 × 10^−5^ and the PI for siblings (PIsib) was 6.4 × 10^−3^, as calculated by the Genalex package. Ninety percent of the fish tested were correctly identified with their respective strains. It is also demonstrated that this panel can be used to confirm doubled-haploid cell lines. This multiplex should find multiple uses for improving the accuracy and reproducibility of studies using the zebrafish model.

## Introduction

Zebrafish (*Danio rerio*) is the vertebrate animal model most rapidly adopted in biomedical research. The zebrafish genome is 70% similar to the human, and 80% percent of the genes responsible for human diseases have an orthologue in zebrafish^1^. It is relatively easy to generate mutant animals, and the cost of zebrafish maintenance is low. Applications of this animal model include, among the many, toxicology, developmental biology, genetics and degenerative diseases, psychiatric conditions, cancer, and metabolic disorders. Researchers can choose from a variety of wild-type and mutant zebrafish lines for their studies. All available from the Zebrafish International Resource Center (ZIRC), wild-type zebrafish lines include Tübingen, AB, NHGRI-1, Sanger AB Tübingen (SAT), WIK, Tüpfel long fin (TL), Nadia, and Cooch Behar. Also available, are more than forty thousand mutant lines.

Despite previous reports of the derivation of the IM inbred zebrafish line in 2011, homozygous zebrafish lines for all loci are lacking^2,3^. Some initiatives are in progress that may fulfill the need for fish lines that have an identical genetic identity. We are currently working on a strategy to generate homozygous diploid fish using the method developed by Streisinger and colleagues in 1981, followed by somatic cell nuclear transfer (SCNT)^4–9^. Once a gynogenetic embryo is developed, we isolate cells from them, and subsequently, after testing for normal karyotype and homozygosity, we generate zebrafish embryos using SCNT. To standardize our genotyping methods, we chose tetra- and penta-short tandem repeats (STRs).

Over 2000 dinucleotide short tandem repeat markers (diSTRs; STRs are also known as microsatellites) have been developed for the zebrafish^10^. Although these markers have proved to be quite useful for zebrafish genetics the diSTRs have some disadvantages compared to STRs with longer repeat motifs^11,12^. The diSTRs have a much higher stutter, an artifact caused by slippage of polymerases used in PCR on the target amplicon^13^. This stutter makes it more difficult to automate genotype calling, particularly for alleles that are a single dinucleotide repeat apart^13,14^. The stutter also “steals” signal from the true allele peak, reducing the ability to call genotypes using low concentrations of DNA that can occur in some samples. In addition, when diSTRs undergo new mutations, about 32% of these new mutations are more than one repeat longer or shorter than the repeat from which they occur, making inferences about kinship more difficult^14,15^. Tetranucleotide and pentanucleotide STRs (hereafter referred to as tetraSTRs and pentaSTRs), on the other hand, appear to follow the “single-step mutation model” much more closely. Only about 1% of new mutations are longer or shorter than one additional or one less repeat, based on human data^15^. Although the data in fish are much more limited, among 17 tetraSTR mutations observed in pink salmon, all were single step, and among 3 mutations observed in diSTRs in common carp, all were multistep^16,17^. We assume that zebrafish will show similar patterns as well, but with the 13-plex detailed in this report the mutation patterns can now be directly addressed.

In human forensics, most STRs have core repeats/motifs that are four or five nucleotides long (e.g., human forensic CODIS markers^18^). These tetra- and pentaSTRs have stutter bands than are generally only about 5–10% of the peak height of the true allele peak compared to the 30% often seen for dinucleotide repeats, making automated allele calling much easier to accomplish for the longer repeat motifs^13^. Here, we report a 13-plex of seven tetra- and six pentaSTRs that should prove useful for a variety of genetic studies in zebrafish, including verification of the correct background line that has been used to produce knock-out and knock-in alleles and, further, we show here that these markers can be used to monitor the production of doubled haploid zebrafish cell lines.

## Materials and Methods

### Fish DNA

Ten fish (five of each sex) were used from each of the wild-type line NHGRI-1, the kca33Tg, and the kca66Tg transgenic reporter lines (these lines were all originally obtained from the Zebrafish International Resource Center [ZIRC] and any family relationships for the individual fish are unknown). DNA samples were taken at our animal facility at MSU. For each line, we used fish belonging to the same generation, 5 males and 5 females. For NHGRI-1 lines we used F0 animals i.e. adult-founders from ZIRC. For Kca33Tg and Kca66Tg lines we used the F2 progeny of founders obtained from ZIRC. DNA was obtained by “scale swabs” from fish present in the laboratory using a modified protocol of previously established methods^19^. Briefly, unanesthetized fish are removed from the tank and placed on a wet sponge or gauze. A plastic-handled cotton swab was then lightly brushed over the scales three times and the fish were returned to the tank. The swabs were then air-dried. The method has been reported to be less invasive compared to tailfin clips and causes less trauma to the fish compared to the use of anesthetics^20^. Dried swabs were stored at room temperature (~20 °C for up to two weeks) if they were not processed immediately, as described below under DNA preparation. Control tailfin DNA was used from previous isolations taken in the lab.

### Homozygous somatic cell lines (HS2) isolation and culture

Diploid gynogenetic embryos were generated as previously described^6,21,22^. Briefly, Metaphase II oocytes were *in vitro* fertilized using sperm derived from transgenic males [Tg(*mylz2*:EGFP)] irradiated with 900 μJ/cm2 UV intensity and used for *in vitro* fertilization. One-cell embryos were incubated at 28.5 °C for 13 minutes, followed by 41.4 °C for 2 minutes and immediately shifted back to 28.5 °C. Embryos were sorted for their morphological differences at 48 hr post fertilization (hpf)^23^. Incomplete diploidization resulted in haploid embryos with abnormal characteristics, while gynogenetic diploid embryos displayed normal development. GFP expression was absent indicating inactivation of the male genome. We then proceeded to isolate fibroblast-like cells from 24 hpf gynogenetic embryos^22^. To this end, single zebrafish embryos were manually dechorionated, euthanized using 0.02% MS222 and bleached in a cold 0.005% solution of bleach in PBS for 5 minutes. Single embryos were then rinsed in PBS three times and transferred to a drop of DNAC medium made of 7% heat-inactivated fetal bovine serum (FBS, Thermo Fisher Scientific, MA), 2% trout serum (Seagrow, EastCoast Bio Inc, CA), 2 mM N- acetyl-L-cysteine (Sigma-Aldrich, MO), 0.2 mM L-ascorbic acid 2-phosphate (Sigma-Aldrich, MO), 50 IU of bovine insulin (Cell Applications Inc, CA), 20 ng/ml of human insulin growth factor (IGF, PeproTech US, NJ)), and 1% 100X Anti-Anti (Thermo Fisher Scientific, MA) in DMEM (Thermo Fisher Scientific, MA)^8^. With the help of 27-gauge needles, single embryos were cut into small tissue pieces that were subsequently placed in Matrigel-coated wells of a 96-well plate containing 50 µl of DNAC medium. Cells were placed in a humidified incubator at 28.5 °C and 5% CO_2_. An equal volume of fresh medium was added 24 hr post plating and half the medium was replaced every 2 days.

### DNA preparation

DNA was prepared using a variation of the “hotshot” procedure^24^. Briefly, after swabbing the fish, the tip of the cotton swab was cut into a 1.7 ml microfuge tube. Four hundred µl of 50 mM NaOH was then added to the tube, and the tube was placed in a Styrofoam floatation device in 70–90 °C water for 5 min. The tubes were uncapped and 40 µl of 1 M Tris (pH 8.0) was added and mixed to neutralize the NaOH. The DNA was then either used immediately or frozen at −20 °C. The HS2 cell line DNA was prepared in essentially the same way, except that 50 µl of cell culture (2.7 × 10^4^ to 1.0 × 10^5^ cells) was first pelleted and resuspended in a 5 µl volume of cell culture medium before the addition of 45 µl of 50 mM NaOH, followed by 5 µl Tris after the high temperature incubation. The final DNA concentration was estimated to be 1.0 to 3.8 ng/µl DNA for the cell lines. Determining the DNA concentration for scale swabs is more difficult using the hotshot method, so we used Nanodrop absorption as a proxie method to provide estimates. The DNA concentration is known for the HS2 cell lines because all manipulations were conducted in a single tube and no material was removed. Under the assumption that the ratio of DNA to the total UV_260_ absorbable material is the same between the HS2 cell lines and the scale swabs, a rough approximation of DNA concentration was calculated.

### Selection of tetra- and pentaSTRs

TetraSTRs and pentaSTRs were initially identified in the 2010 build of the zebrafish reference genome (Zv9/danRer7). Within this older build is a version of RepeatMasker that has Smith-Waterman scores for STRs that can be used to predict the expected heterozygosity of STRs for a given effective population size with reasonable accuracy *(Venta, unpublished results)*. Briefly, tetraSTRs and pentaSTRs with SW scores between 450 and 700 were initially identified as likely to have good expected heterozygosity but relatively low mutation rates compared to markers with scores above 700. These potential markers were then inspected for areas of unique sequence flanking the STRs (i.e., those without other repetitive elements such as the retrotransposon SINEs or LINEs [short and long interspersed nuclear elements]) and with the longest uninterrupted sequences (LUS; also known as “perfect repeats”), being from 8 to 16 repeats; the lower limit was to ensure variability and the upper limit was to minimize the height of stutter peaks^25^. Markers were also selected from separate chromosomes to assure independent assortment.

### Primer design

Primers for the STRs that met the criteria mentioned in the previous section were then designed using the online Primer3 tool (http://bioinfo.ut.ee/primer3-0.4.0/). In order to facilitate multiplexing, most primers were chosen that ended in at least two adenines (AA). This method, originally developed more than twenty years ago, helps reduce complementarity among primers at the 3′ end, which is believed to be involved in primer-dimer formation^26,27^. We have used the method to develop a 12-plex for jaguars, a 12-plex for domestic pigs, a 12-plex for honeybee, and a 16-plex for STRs near genes that, when mutant, can cause blindness in dogs^28,29^ (*and Venta unpublished results*). The method was also used in part to produce a 17-plex of tetraSTRs for horses *(Venta unpublished results)*. Primers were also checked for compatibility by AutoDimer^30^ (https://www-s.nist.gov/dnaAnalysis/index.do). A short 7 base sequence (GTTTCTT, called a “pigtail”) was added to the 5′ end of all reverse primers to suppress “peak splitting” due to the terminal transferase activity of the Taq DNA polymerase^31^.

### Universal primers

A universal primer system was used for developmental validation purposes, as well as for the completed multiplex. The four universal primers used here also end in AA to suppress the production of primer-dimer formation^28,29^. Additional design rules used to produce these universal primers are given in Corner *et al*., 2018. For convenience, the sequences for the universal primers are listed here: uni-1, CTCCAACTCACCTCCAACAAA; uni-2, AAACCTCTCTCCACACCCAAA; uni-3, CTCACCTCCCACTCCACAAA; and uni-4, AACTCCACCACTCCCACAAA. The “uni” primers were randomly assigned fluorescent dyes, as shown in Table 1 (6-FAM-labeled primer from IDT through IDTdna.com; and NED-, PET-, and VIC-labelled primers from ABI, obtained through Thermo Fisher Scientific).

**Table 1:** Zebrafish tetra- and pentaSTR locus and primer information.

locus	start	primer sequence	motif	SWS	perf rep	ref+tails	uni-dye	µM
chr23	1341696	F: AATATGTGACGTAGGTAGTAATGCAG	AATA	456	12	127	uni1-FAM	0.020
		R: TGCATTTGCTTATGTTTTTCttg						0.197
chr15	2218411	F: GAAGCCTGGGTCATTTTCAG	AAAC	534	11	147	uni2-PET	0.059
		R: GAAAATTCCTAAACTTTTATTCAGGTAGAA						0.592
chr17	2618753	F: gaggatgaaagcaGCACTTAAAA	ATTC	549	15	152	uni3-NED	0.040
		R: TGACAAATTGGAGAGTTGATTAAA						0.395
chr22	1488958	F: GAAGTCCTTACCAGCCACAAA	ATAG	594	16	167	uni4-VIC	0.010
		R: TTTAAAAGGTCAACAACGCATA						0.099
chr11	2435143	F: CGTGGCAGGAGCTGTGAA	TATTA	558	12	212	uni4-VIC	0.027
		R: CGCATTATGCCAGTCATGATAA						0.267
chr19	75036748	F: TTTGGTAATTTGATGATTTGGAA	TATAT	558	12	236	uni1-FAM	0.013
		R: TGTTAACGTCATAAAAATCTGATAAA						0.133
chr24	7379615	F: ACATTCTGGCTCCCATCAGA	ACTAC	561	11	238	uni3-NED	0.013
		R: CAGGCCTCTTGTGTTTGTGA						0.133
chr05	1147730	F: GCGGACATCTGACAACTGAA	ATAA	513	15	253	uni2-PET	0.053
		R: GGATGTGTGAAACGCAGATAA						0.533
chr14	2009429	F: AAGTGCAGCAGAGAATGTTGTTA	GATG	552	8	276	uni3-NED	0.020
		R: GCTGAGGCACGTATTTCCAA						0.200
chr21	2820816	F: GTCTCTCACCTCGTCTGTGAA	AAAC	537	11	319	uni1-FAM	0.020
		R: GGCTGTAAACATGAGCGAAG						0.200
chr04	1653932	F: GAGGATCGGAAAGCAGGTAAA	AATAC	522	11	375	uni1-FAM	0.013
		R: TGTTTGACTCAACTGTGGGTAA						0.133
chr09	2073768	F: tatataAAACACTATGTATTTGTATAATCAGAA	ATATA	522	11	419	uni4-VIC	0.027
		R: GACAAGACGGCTTCAGCAA						0.267
chr02	2168981	F: CCAACATCCGGCACAAATAA	TATTA	553	11	442	uni2-PET	0.040
		R: ATGTCTGAGGAAATATGAAATGATGAATAA						0.400

This table is sorted by increasing size of the amplicon. The locus name is given as the chromosome upon which it is located, followed by the genome position number of the simple tandem repeat (start; beginning position of the repeat from the zebrafish reference genome build Zv9). All F (forward) primers are labeled at the 5′ end with one of the universal primer tags/tails (sequence not shown), and all R (reverse) primers include a pigtail sequence (GTTTCTT; not shown) at the 5′ end to suppress the production of split peaks. Lower case letters within the primer sequences indicate repeats (e.g., SINEs or LINEs) over which the 3′ end of a primer may be partially designed. The motif for the tetra- and pentaSTRs is given in the next column and it is based upon the output of the Tandem Repeat Finder (TRF) in the UCSC Genome Browser. The Smith-Waterman score (SWS) provides an indication of the expected heterozygosity (discussed in the Materials and Methods section). The perfect repeat number (perf rep) is shown because it is correlated with the amount of stutter produced for a given STR^25^. The reference genome size for the PCR product is given with the uni-primer and pigtail attached (ref+tails). The universal primer (uni-dye) information is given in the Methods and Materials section. The final concentration for each of the locus-specific primers is given in the last column. The final concentration of each universal primer is: uni-1, uni-3, and uni-4, 0.011 µM; and uni-2, 0.043 µM.

### Genotyping

Primer sequences and final concentrations for the multiplex are listed in Table 1. Primer concentrations were titrated to produce relatively even amplifications among the STRs. DNA samples were amplified under the following conditions: during the development of individual markers for the panel, 50 mM KCl, 10 mM Tris (pH 8.3 at 20 °C), 2.0 mM MgCl_2_, 100 µM dNTPs, primer concentrations as in Table 1, 1 µl of DNA per 25 µl reaction, and 0.04 U Ampli-Taq Gold/µl, or, for the complete multiplex panel for genotype analysis, by using the Qiagen Multiplex Type-It Microsatellite reagent in 5 µl reactions. PCR times and temperatures were as follows: 5 min at 95 °C, then 50 cycles at 1 min 94 °C, 2 min 57 °C, and 3 min at 72 °C, with a final cool-down to 20 °C for 20 min. Initial assessment of amplification was made by 2% agarose gel electrophoresis, followed by scanning with a Typhoon FLA 9500 Scanner (G.E. Corp.) using Cy2 (for FAM and VIC) and Cy3 (for NED, PET, and VIC) filter settings, followed by staining with ethidium bromide and UV photo-documentation to obtain compare to unlabeled size standards. Two µl of multiplexed PCR product was directly added to 20 µl of deionized water, and high-resolution genotyping was performed on an ABI 3730xl machine at the MSU Research Technology Support Facility. Genotyping calls were made using Peak Scanner software (ABI) on the .fsa output files.

### Determination of population genetic parameters

Rounding errors in bp were manually corrected before the genotyping data was analyzed with the Genalex analysis package^32,33^. Observed and expected heterozygosities (Ho and He, respectively) were calculated by Genalex, as well as identity indices and inferences concerning the relatedness of the lines that were genotyped.

All animal experiments were approved by the Michigan State University IACUC. All animal experiments were carried out in accordance with relevant guidelines and regulations described in ‘The Zebrafish Book’ and by ZM Varga 2016^22,34^.

## Results

### General observations

A zebrafish 13-plex was developed that consists of seven tetraSTRs and six pentaSTRs (Table 1). All STRs gave reasonably strong and even amplification under the conditions used (discounting a few null alleles in some samples). Large amplicons had smaller peak heights than smaller amplicons for scale swab DNAs, but these peaks were still callable. Higher quality DNA (tailfin DNA) had more uniform peak heights among all amplicons (Supplementary Fig. S1). Although the DNA yield for zebrafish scale swabs has previously been previously reported, we provide a crude estimate here based on the proxie method described in the methods section^24^. DNA yields ranged from 74 to 192 ng (mean 114 ± 27), which appears to be somewhat lower than the original scale swab report (~300 ng, assuming some RNA contamination in that report). All of the fish examined had unique genotypes. The observed and expected heterozygosities for the three fish lines examined are given in Table 2. The average observed heterozygosity (Ho) and average expected heterozygosity (He) for all fish among the three lines examined were 0.291 and 0.359, respectively. Stutter bands for the alleles of all markers were less than 14% of the main peak (the highest value is for the longer of the alleles of marker chr22, which has the longest reference genome perfect repeat of any of the markers; Table 1). Allele sizes for any given marker were generally distributed in a discontinuous fashion, an observation considered to be prima facie evidence of a dramatic reduction an effective population size^35^ (Supplementary Table S1).

**Table 2:** STR data for three zebrafish lines for the 13-plex.

locus	alleles	range	NHGRI-1		kca33Tg		kca66Tg	
			Ho	He	Ho	He	Ho	He
chr23	4	113–144	0.100	0.375	0.900	0.565	0.000	0.000
chr15	4	102–113	0.400	0.480	0.400	0.460	0.333	0.494
chr17	2	109–144	0.000	0.000	0.100	0.095	0.000	0.000
chr22	2	124–156	0.600	0.480	0.600	0.420	0.400	0.420
chr11	4	206–239	0.100	0.095	0.500	0.665	0.300	0.265
chr19	4	241–258	0.000	0.219	0.700	0.515	0.750	0.594
chr24	5	227–242	0.000	0.494	0.000	0.000	0.000	0.000
chr05	7	144–240	0.800	0.665	0.200	0.180	0.100	0.345
chr14	4	258–276	0.400	0.320	0.200	0.180	0.333	0.364
chr21	4	299–317	0.700	0.540	0.300	0.255	0.000	0.000
chr04	3	360–384	0.300	0.535	0.000	0.568	0.200	0.700
chr09	6	408–454	0.300	0.635	0.222	0.747	0.250	0.758
chr02	5	399–499	0.000	0.000	0.500	0.465	0.111	0.105

Allele counts, observed heterozygosity (Ho) and expected heterozygosity (He) were obtained using Genalex with the data in Supplementary Table S1 after correction of allele rounding errors. Markers are sorted in the same order as in Table 1.

### Single alleles for all markers in the HS2 cell lines

The HS2 cell lines showed only a single allele for all markers that produced observable results (with an average of 12 markers scored per cell line; Supplementary Table S1). For all of the cell lines and all of the markers, only one or two alleles were observed across all ten cell lines because each of these cell lines were derived from a gynogenetic fish. Only a single fish (kca66Tg-4) from the lines showed homozygosity for all twelve markers that amplified for this sample (one marker was presumed to be homozygous for a null allele; Supplementary Table S1). All other zebrafish had at least one heterozygous marker, with an average of 3.6 heterozygous markers (and 8.4 homozygous markers) per fish.

### Observations on population genetic parameters

The probability of identity (PI) for all fish tested was 2.1 × 10^−5^. For the individual lines these values were: NHGRI-1(1.7 × 10^−5^), kca33Tg (4.1 × 10^−6^), and kca66Tg (3.9 × 10^−5^). The PI_sib_ was lower, as expected (6.4 × 10^−3^), but it is still quite useful. Using Genalex, all 30 fish were correctly assigned to their line except for three: one fish, kca33Tg-3, clustered with kca66Tg population, and two fish, kca66Tg-2 and kca66Tg-5, clustered with kca33Tg population.

### Null alleles

Null alleles were seen in seven of the 13 markers in at least one zebrafish line. Three of these nulls occurred in markers chr04, chr15, and chr21 which occurred in only a single fish, kca66Tg-6, which may indicate that the DNA of this fish is of lower quality than the other samples (i.e., lower concentration and/or shorter DNA fragment sizes) (Supplementary Table S1). Kca66Gt-6, in fact, had the lowest amount of UV_260_ absorbable material among all the zebrafish scale swabs. The speculation of lower quality DNA for this scale swab is also partially supported by the fact that four of the markers that failed to amplify are the largest ones (all greater than 300 bp) although the other two markers are of a similar size to the ones that did amplify. It is possible that these two amplify somewhat less efficiently that the other markers and, in combination with a lower quality DNA sample, may have for this reason failed to amplify at all. One marker, chr19, had null alleles across two lines (kca66Tg-6 was also null for this marker, although we note above that it may be poorer quality DNA), and two markers, chr05 and chr09, had null alleles across all three zebrafish lines (Supplementary Table S1). These three markers are still useful for individual and line identification purposes. During the development of this panel, many markers were set aside because of the occurrence of null alleles, which is at a higher frequency from the experience we have had with all of the other species for which we have produced markers to date (e.g., jaguars and pigs)^28,29^. The primers for the markers with nulls, which may still be useful in other lines, are given in Supplementary Table S2.

## Discussion

### Development of markers and comparison to previous work with diSTRs

A tetra- and pentaSTR 13-plex has been developed for zebrafish. Markers with longer motifs are known to be easier to genotype and to allow more accurate inter-laboratory comparisons^13,14^. This 13-plex should also result in lower costs for resources and time because all markers are amplified simultaneously. Although the heterozygosity for these markers is lower than 50%, this is likely due to the fact that zebrafish lines are bred in such a way that they have low effective population sizes. Genetic drift will have removed many alleles for each marker, resulting in reduced heterozygosity^35^. Previous observations with these types of markers in other species (including other fish species) suggests that the markers in the 13-plex should show an average above 90% of the maximum value for heterozygosity for markers with similar Smith-Waterman score values^28,29^ (*and Venta unpublished results*).

Low heterozygosity in zebrafish diSTRs has been seen by other researchers for zebrafish lines, as well^36^. In fact, Coe *et al*. also examined wild caught zebrafish from Bangladesh, in which case their four diSTRs showed an average observed heterozygosity of 0.743 and an average expected heterozygosity of 0.855^36^. We have left out their marker Ztri1 in this calculation, because this is a long tetraSTR (major motif is AATG), based upon the sequence in the reference genome, but the result when this repeat was included was nearly identical (Ho = 0.714 and He = 0.855). Coe and colleagues also found that fish designated as one line sometimes clustered with other lines, as we have found^36^. They suggested that this was due to cross breeding of the lines. This is consistent with our results, as well. For example, the 149 bp and 166 bp alleles are the only two alleles seen in all three lines examined in this study (Supplementary Table S1), whereas long tetraSTRs in outbred species tend to have a continuous distribution of allele sizes. Although we used only moderate sample sizes, given the likely low effective population sizes for these lines, they should be adequate to capture most of the common alleles in the sample^35^. Accurate effective population size is a notoriously difficult value to estimate, but based upon estimations in other species and associated heterozygosity, we speculate that inbred zebrafish lines have effective population sizes below 100, whereas wild populations have effective sizes in the thousands^35,36^.

### Fish scale swabs

Fish scale swabs are a convenient way to obtain DNA samples that are less invasive to the fish^20^. Although larger amplicons do not amplify as well with fish scale DNA as with whole tissue (such as tailfins) DNA, they are callable, and scale swabs have other useful advantages. We speculate that the average genomic DNA fragment size is shorter due to apoptotic DNA degradation in loose scales, but further experiments will be needed to test this hypothesis. Once the samples have been air-dried, they are stable for at least two weeks at room temperature. Our experience with canine cheek swab suggests that they will be stable for much longer, because we have stored these swabs for more than ten years at room temperature, with no obvious degradation in PCR amplification properties. Samples prepared in this manner can easily be sent by mail, which is the standard practice among canine genetic testing companies. Stability studies with scale swabs of zebrafish has only begun, but we cannot foresee any reason that the results will be any different from that for dog samples. In this report, we have used cotton swabs which we compared to the Dacron swabs used in the original zebrafish hotshot study^19^. We did not detect any difference in our ability to call genotypes due to the difference in swab material *(unpublished results)*.

### High frequency of markers with null alleles

Although null alleles are eventually found for virtually any genetic marker in any species, the frequency of markers with such nulls seems to be higher in this study of zebrafish than for other studies of zebrafish or most other species^37^. Published reports on zebrafish diSTRs have mentioned null alleles (in 3 to 5% of the diSTRs examined), but we have found in this work that this is a more significant problem (35% among the total number of tetraSTRs examined in this report; Table 1 and Supplementary Table S2) than reported in these earlier papers^10,38^. We do not know if the reason for the difference in the observation in this report vs. the previous reports is due to some phenomenon related to the repeat type (diSTRs vs. tetraSTRs), the particular zebrafish samples examined, or for some other reason. We redesigned R primers for three tetraSTRs that had high frequency of null alleles among the HS2 cell lines, but these markers still did not amplify among the samples originally used to identify the nulls. Markers with high frequency null alleles were dropped from the final multiplex. Although the presence of null alleles is unlikely to present misidentification problems, for population studies the markers with nulls should be monitored carefully or perhaps even set aside. Primer sets that had high null allele frequencies that were not included in the 13-plex are given in Supplementary Table S2.

### Use of the 13-plex to verify homozygosity in double haploid cell lines

All markers that produced peaks were homozygous among the HS2 doubled haploid cell lines. Although we have not formally shown here that this is not due to true haploidy as opposed to diploidy/doubled haploidy, based upon the method used to produce these cell lines, which has similarities to other doubled haploid fish technologies, these cell lines are also highly likely to be doubled haploid in nature.

### Further uses of the zebrafish 13-plex

In addition to monitoring production of doubled haploid cell lines, this 13-plex can be used for a variety of other purposes in zebrafish studies. In mammalian studies it has been estimated that up to 30% of publications using cell lines, those cell lines have been mis-identified, leading to uncertainty and irreproducibility in the literature^38,39^. This 13-plex should be useful for verifying the identity of zebrafish lines and cell lines. Paternity is another important use for identifying parameters that affect social behavior, such as competition in reproduction^36^. The multiplex may also find use for studies of cancer in which elevated microsatellite alterations at selected tetranucleotides (EMAST) have been shown to occur^40,41^. These are just a few examples of how this zebrafish 13-plex may be of use beyond our primary goal of using the markers for verification of doubled haploid cell lines.

### Summary and conclusion

Although SNPs are now the most important type of marker for many purposes in genetics, STRs are quite often more cost effective, are simpler to use, and generally require less complicated software to reach valid conclusions. Although care should always be taken in selecting a particular marker type, we believe this 13-plex zebrafish panel will be excellent tool for many researcher purposes^42^.

## Supplementary information

Supplementary information.

Supplementary information 2.

Supplementary information 3.
