# Supplementary material for: A 13-plex of tetra- and penta-STRs to identify zebrafish
Source: Sci Rep. 2020 Mar 2;10:3851. doi: 10.1038/s41598-020-60842-5 (PMC7052278; doi:10.1038/s41598-020-60842-5)
Supplement: Supplementary file 1 — Supplementary information. [file 41598_2020_60842_MOESM1_ESM.pdf]

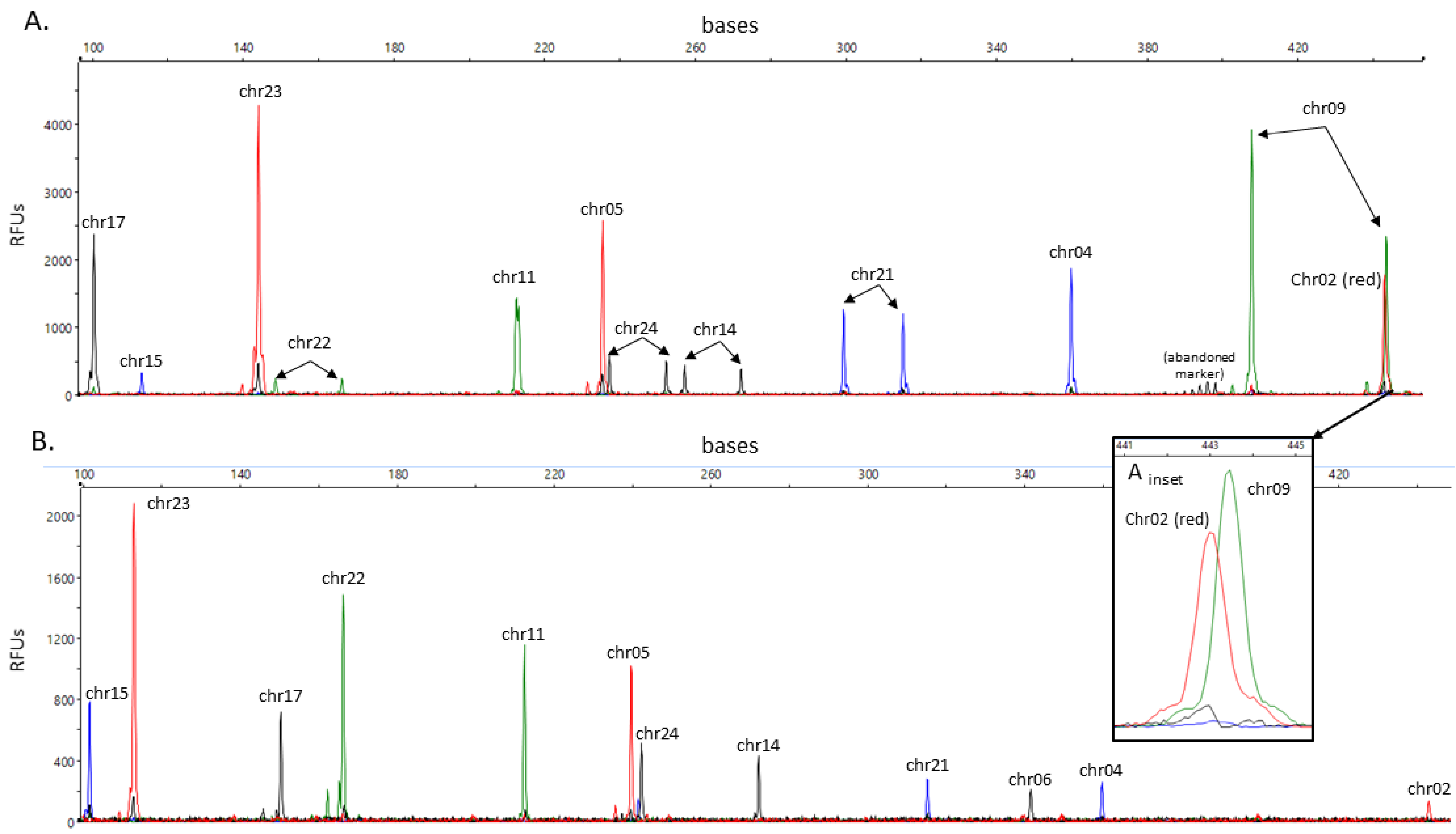


**Supplemental Figure S1**: **Electropherograms of the zebrafish 13-plex in which a comparison is made between a heterozygous zebrafish and a doubled haploid cell line.** A. This panel shows the result for zebrafish NHGRI-1-1 that was amplified from DNA purified from a fin. Note that five markers (chr04, chr14, chr21, chr22, and chr24) show heterozygosity. Two markers (chr06 and chr19) failed to amplify in this fish, and a dinucleotide stutter (due to a previously unidentified [CA]9 in the amplicon) can be seen for an abandoned marker at about 390 bases. The abandoned marker is no longer in the panel. RFUs are relative fluorescence units. The A inset expands the chr02 and chr09 peaks to make it easier to distinguish them. B. This panel shows the result for the HS2-3 doubled-

haploid cell line in which all markers show only a single allele. Two markers did not amplify (chr09 and chr19) because of null alleles in this sample. Scale swabs have peak heights (data not shown) similar to the cell line in panel B for which the DNA was not extracted from the cells until several hours after they were harvested and are thought to have undergone some apoptosis reducing the average size of the genomic DNA fragments. Note that the peak heights across marker amplicons are more uniform in panel A compared to panel B. For example, the height of the peak for chr02 near the right edge of the figure is more similar to height of the chr23 marker in panel A compared to the same marker peak heights in panel B.
